# Supplementary material for: Distinct roles of striatal direct and indirect pathways in value-based decision making
Source: eLife. 2019 Jul 16;8:e46050. doi: 10.7554/eLife.46050 (PMC6658164; doi:10.7554/eLife.46050)
Supplement: Supplementary file 1. [file elife-46050-supp1.docx]

**
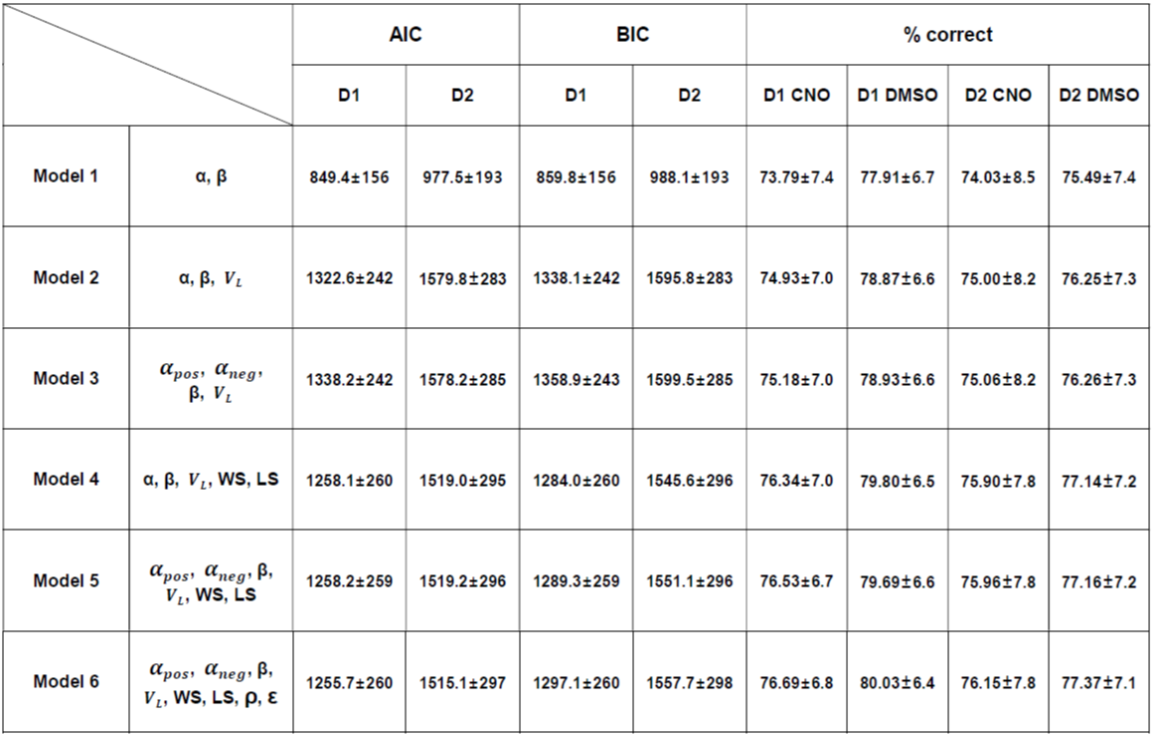
**

**Model comparison.** Akaike’s information criteria (AIC), Bayesian information criteria (BIC), and the accuracy to predict the animal’s actual choice (leave-one-out cross-validation; % correct) in the TAB task are shown for different models. Mean±SEM across animals.
